# Community-Based, Point-of-Care Sexually Transmitted Infection Screening Among High-Risk Adolescents in Los Angeles and New Orleans: Protocol for a Mixed-Methods Study

**DOI:** 10.2196/10795

**Published:** 2019-03-22

**Authors:** Chelsea Lee Shannon, Maryann Koussa, Sung-Jae Lee, Jasmine Fournier, Sue Ellen Abdalian, Mary Jane Rotheram, Jeffrey D Klausner, Adolescent Medicine Trials Network CARES Team

**Affiliations:** 1 Division of Infectious Diseases David Geffen School of Medicine University of California Los Angeles Los Angeles, CA United States; 2 Semel Institute for Neuroscience and Human Behavior University of California Los Angeles Los Angeles, CA United States; 3 Section of Adolescent Medicine Department of Pediatrics Tulane University New Orleans, LA United States

**Keywords:** sexually transmitted infections, adolescents, point-of-care testing

## Abstract

**Background:**

Sexually transmitted infection (STI) rates are increasing in the United States, with approximately half of new infections occurring among adolescents aged 15-24 years. Gay, bisexual, and transgender youth (GBTY), homeless youth, and youth with histories of drug use, mental health disorders, and incarceration are all at uniquely high risk for STIs. However, these adolescents often lack access to sexual health services.

**Objective:**

This study aims to use point-of-care STI tests in community-based settings to screen for and treat STIs in adolescents.

**Methods:**

We are recruiting 1500 HIV-uninfected youth and 220 HIV-infected youth from homeless shelters, GBTY organizations, and community health centers in Los Angeles, California and New Orleans, Louisiana. Study participants will receive STI screening every 4 months for 24 months. STI screening includes rapid HIV, syphilis, *Chlamydia trachomatis, Neisseria gonorrhoeae,* and Hepatitis C virus testing. Trained paraprofessionals will conduct all STI testing. When a participant screens positive for an STI, they are either linked to a partner medical clinic or provided with same-day antibiotic therapy and expedited partner therapy. We will monitor STI prevalence among study participants as well as point-of-care test performance, linkage to care, and treatment outcomes.

**Results:**

The project was funded in 2016, and enrollment will be completed in 2019. Preliminary data analysis is currently underway.

**Conclusions:**

As STI rates continue to rise, it is important to improve access to screening and treatment services, particularly for high-risk adolescents. In this study, we aim to evaluate the use of point-of-care STI diagnostic tests in community-based organizations. We hope to determine the prevalence of STIs among these adolescents and evaluate the acceptability and feasibility of community-based STI screening and treatment.

**Trial Registration:**

ClinicalTrials.gov NCT03134833; https://clinicaltrials.gov/ct2/show/NCT03134833

**International Registered Report Identifier (IRRID):**

DERR1-10.2196/10795

## Introduction

**Figure 1:**
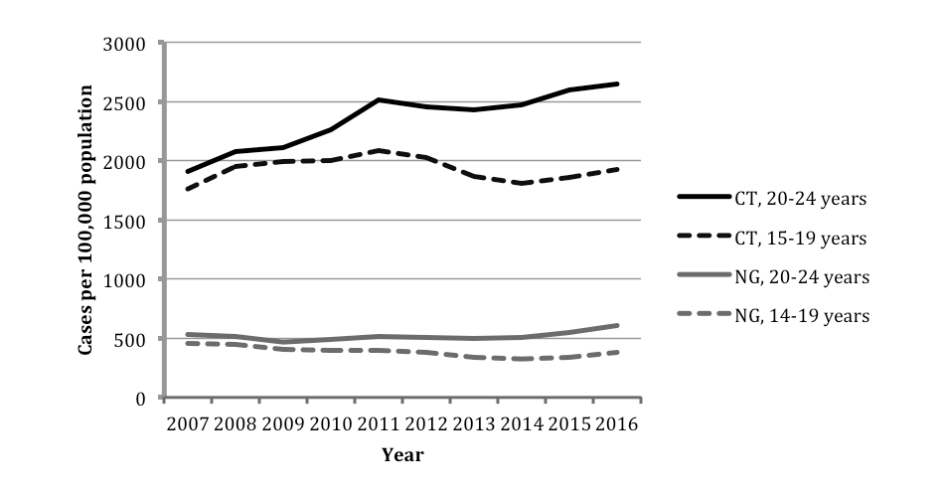
*Chlamydia trachomatis* (CT) and *Neisseria gonorrhoeae* (NG) infection rates among adolescents from 2007 to 2016.

There are approximately 20 million new sexually transmitted infections (STIs) every year in the United States. Half of these infections occur among adolescents aged 15-24 years [1]. STI rates have been steadily increasing over the past few years, with adolescent rates of *Chlamydia trachomatis* (CT) infection, *Neisseria gonorrhoeae* (NG) infection, and syphilis infection on the rise (Figure 1) [2,3].

Adolescents are at particularly high risk for STIs due to a combination of behavioral, biological, and social factors. Behaviorally, adolescents are more likely to engage in higher-risk sexual behaviors such as concurrent partners or sex without a condom. Biologically, adolescent females are often more susceptible than adult women to contracting an infection if exposed [2,4]. Socially, adolescents often lack access to sexual health services or do not pursue STI testing due to confidentiality concerns [5].

STI prevalence is highest in the southern and western United States, with black and Latino adolescents at particularly high risk [2]. Social and geographic differences in STI prevalence are likely due to systemic inequalities leading to limited access to sexual health services and reduced rates of STI screening [6]. The stigma surrounding sexual health may also contribute to reduced screening uptake. Gay, bisexual male, and transgender female youth are at an increased risk for STIs due to a combination of risk factors, such as condomless sex, concurrent partners, and sex with older partners [2]. Receptive anal intercourse also has a higher STI transmissibility than other forms of intercourse. Among gay, bisexual, and transgender youth (GBTY), parental rejection, stigma, discrimination by peers, and increased stress associated with being a member of a minority, whether that minority status comes from race, ethnicity, socioeconomic status, or sexual orientation, may increase sexual risk-taking behaviors and STI rates. Finally, several studies have shown that homelessness, a history of incarceration, and illicit drug use are also associated with increased STI risk in adolescents [7-9]. Reduced access to sexual health services, high levels of stigma, and increased rates of risk-taking behaviors may all contribute to lower rates of screening and higher prevalence of STIs in these populations.

It is critical to diagnose and treat adolescent STIs for a number of reasons. Left untreated, many STIs can lead to long-term health consequences. Bacterial STIs such as CT and NG may lead to reproductive system damage, while syphilis can cause serious neurological damage [10-12]. Viral STIs such as human papillomavirus, herpes simplex virus, and hepatitis C virus (HCV) can cause cancer, genital blisters, and liver failure, respectively [13-15]. Furthermore, STIs increase the risk of acquiring HIV infection 3-fold to 5-fold [16].

Fortunately, diagnostic tests are available for many STIs. Specifically, rapid diagnostic tests create a new opportunity to screen for and treat STIs in community-based settings previously unequipped to offer testing services [17-19]. As these tests become more readily available, it is important to understand their effectiveness in diagnosing and treating STIs in high-risk adolescent populations. By understanding this, we can better evaluate if rapid STI testing in community-based settings may help improve access to sexual health services, reduce stigma, and prevent confidentiality concerns among key high-risk populations.

In this component of the Comprehensive Adolescent Research and Engagement Studies (CARES), part of the Adolescent Medicine Trials Network (ATN) for the HIV/AIDS Interventions Research Program Grant (National Institutes of Health grant U19HD089886), we aim to evaluate the use of rapid STI testing among adolescents at community-based organizations in Los Angeles, California and New Orleans, Louisiana. Rapid STI testing will be administered to 1500 high-risk HIV-uninfected youth and 220 HIV-infected youth every 4 months over the course of 2 years. We will monitor STI prevalence, acceptability and feasibility of rapid diagnostic STI testing, and STI treatment outcomes.

## Methods

### Objectives

We are conducting rapid HIV, CT, NG, syphilis, and HCV testing among adolescents aged 15-24 years at community-based organizations in Los Angeles and New Orleans. Our partner community-based organizations cater to GBTY, homeless youth, youth with a history of mental health disorders, and youth with a history of incarceration. Study participants receive STI testing at 4-month intervals for 24 months, totaling 1 baseline visit and 6 follow-up visits. When a participant receives a positive STI test result, they are linked to care at a nearby medical clinic or provided with antibiotic treatment by the interviewing staff. We hypothesize that by providing rapid STI testing among high-risk adolescent populations, we will find STI prevalences higher than the national averages for adolescents. We intend to evaluate the acceptability of testing as the uptake of screening among eligible participants, and we intend to evaluate the feasibility of treatment as the proportion of participants who test positive for an STI and receive treatment. We expect that by providing same-day testing results and treatment, we will be able to provide quicker time to treatment and higher treatment rates than with traditional lab-based testing. We will compare our time to treatment and treatment rates with historical data from AIDS Healthcare Foundation, Los Angeles. We also intend to evaluate other treatment outcomes such as cure rates, reinfection rates, and partner treatment rates. For objectives of other components of the ATN CARES study, refer to other ATN CARES protocol papers [20-24]. For specific power analyses, refer to the ATN CARES paper by Swendeman et al [22].

### Research Ethics and Approval

The Institutional Review Board of the University of California, Los Angeles has approved the study protocol (16-001674-AM-00006). We will report any protocol deviations or indications of adverse events to the Institutional Review Board. The study was registered on ClinicalTrials.gov (NCT03134833) on April 28, 2017.

### Sexually Transmitted Infection Tests

We selected rapid STI tests according to performance, availability, and cost. Table 1 shows the sensitivity and specificity values of each test.

**Table 1:** Sensitivities and specificities of sexually transmitted infection rapid diagnostic tests.

Test name			Sensitivity (%)	Specificity (%)
Determine HIV-1/2 Ag/Ab Combo [25]			99.9	99.8
Xpert HIV-1 Qual [26]			98.7	99.9
Syphilis Health Check [27]			71.4	91.5
Hepatitis C Virus Rapid Antibody Test [28]			99.9	99.9
**Xpert CT^a^/NG^b^ Assay [29]**				
	**Vaginal swabs**			
		CT	99.5	99.1
		NG	99.9	99.9
	**Urine**			
		CT	98.5	99.8
		NG	98.3	99.9

^a^CT: *Chlamydia trachomatis.*

^b^NG: *Neisseria gonorrhoeae.*

**Figure 2:**
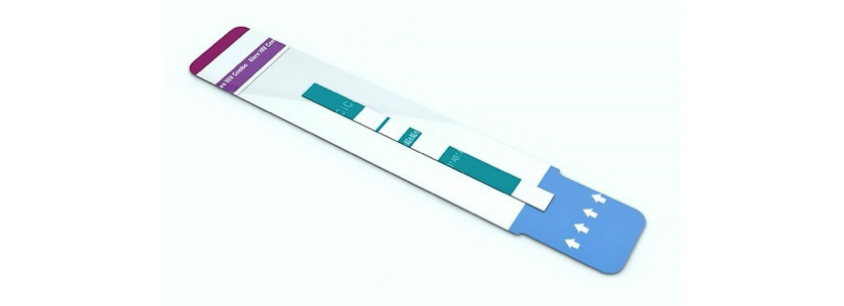
Determine HIV-1/2 Ag/Ab Combo test.

HIV antigen and antibody screening are done using the Determine HIV-1/2 Ag/Ab Combo test (Alere Inc) (Figure 2) [30]. This test is a point-of-care lateral flow strip that detects both HIV-1 and HIV-2 antibodies and the HIV-1 p24 antigen using 50 µL of fingerstick whole blood. The window period is 12-26 days, and results are ready in 20-40 minutes. The test is Clinical Laboratory Improvement Amendments (CLIA) waived and Food and Drug Administration (FDA) approved [25,31].

**Figure 3:**
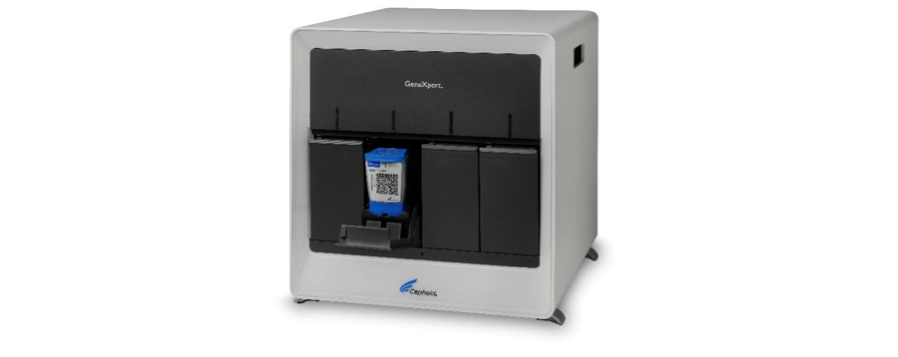
GeneXpert machine used for HIV-1 Qual and CT/NG tests.

We will perform HIV RNA and DNA screening with the Xpert HIV-1 Qual test (Cepheid) (Figure 3) [32]. The test is a point-of-care qualitative *in vitro* HIV test, detecting HIV-1 RNA and DNA. The HIV-1 Qual test requires 100 µL of whole blood, and results are available in 90 minutes [26]. The test is approved for use in the European Union and undergoing the approval process with the FDA. Our study is the first in the United States to use the test, and results are available as research use only.

The HIV-1 Qual test can detect HIV infection an average of 5 days earlier than a p24 antigen test. Therefore, this test is done to detect acute HIV infections that we may not be able to detect with the Alere HIV test.

**Figure 4:**
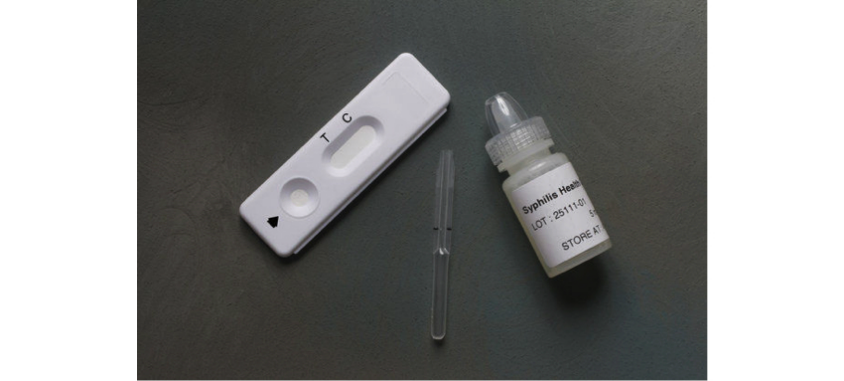
Syphilis Health Check test.

Syphilis screening is done using the Syphilis Health Check, a rapid point-of-care treponemal antibody test (Diagnostics Direct) (Figure 4) [33]. The test uses 50 µL of whole blood, and results are available in 10 minutes. The Syphilis Health Check is the only FDA-approved rapid syphilis test [27].

**Figure 5:**
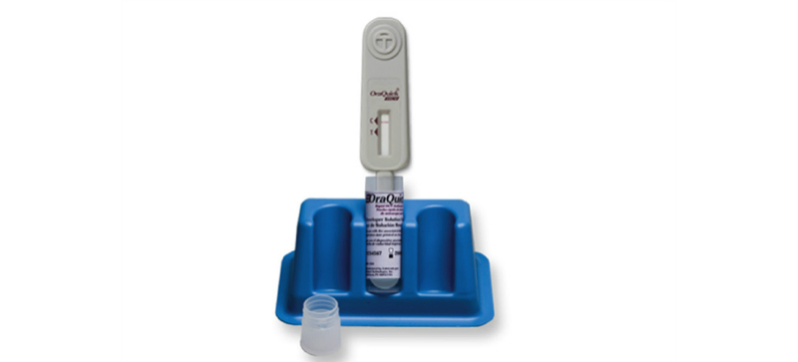
HCV Rapid Antibody Test.

We will perform HCV screening with the HCV Rapid Antibody Test (OraSure Technologies), a rapid point-of-care assay used for the detection of HCV antibodies (Figure 5) [34]. The test uses whole blood and gives results in 20-40 minutes. The test has a waiver from the CLIA and is approved by the FDA [28].

Finally, we will perform CT and NG screening using the Xpert CT/NG Assay (Cepheid) (Figure 3). The test is a qualitative *in vitro* real-time polymerase chain reaction test for the detection of CT and NG. Results are available in 90 minutes [29]. The test is FDA approved for urine samples and vaginal swabs. However, it is also verified in accordance with CLIA for pharyngeal and rectal swabs [35]. Male participants self-collect pharyngeal and rectal swabs as well as a urine sample, while female participants self-collect pharyngeal, rectal, and vaginal swabs.

### Training

Interviewing staff conducts all STI and HIV rapid testing at community-based recruitment sites. Interviewers are typically Bachelor of Arts-level paraprofessionals with little prior experience related to rapid diagnostic testing. Some have previously received phlebotomy training, but most receive phlebotomy training upon hiring. Interviewers receive training and certification in state-specific HIV counselor training. HIV counselor training includes training on fingerpricking, conducting different types of rapid HIV tests, interpreting results, and providing counseling regarding safe sex practices. We also coordinate specific training in Los Angeles and New Orleans for each diagnostic test. The respective diagnostic test companies (Alere, Cepheid, Diagnostics Direct, and OraSure) conduct the training. We evaluate interviewers on their ability to properly collect fingerprick blood and on their ability to correctly interpret test results. We conduct repeat diagnostic test training every 6 months to ensure interviewers continue to correctly perform tests. A binder with step-by-step test instructions is at every site in case any questions arise.

**Figure 6:**
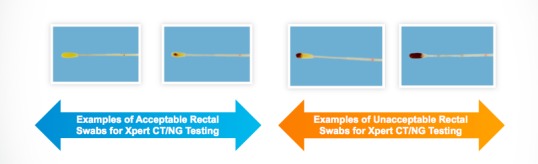
Instructions for self-collected rectal swabs. CT: *Chlamydia trachomatis*; NG: *Neisseria gonorrhoeae*.

We also train interviewers on how to instruct participants to self-collect rectal swabs, pharyngeal swabs, vaginal swabs, and urine samples. For rectal swabs, we provide an image to show the acceptable level of fecal contamination on the swab (Figure 6).

**Figure 7:**
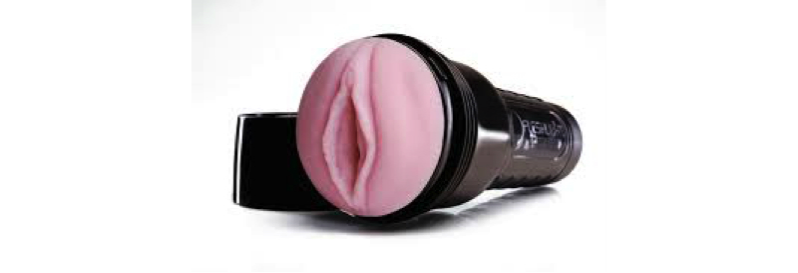
Vaginal Fleshlite used to demonstrate how to self-collect vaginal swabs.

**Figure 8:**
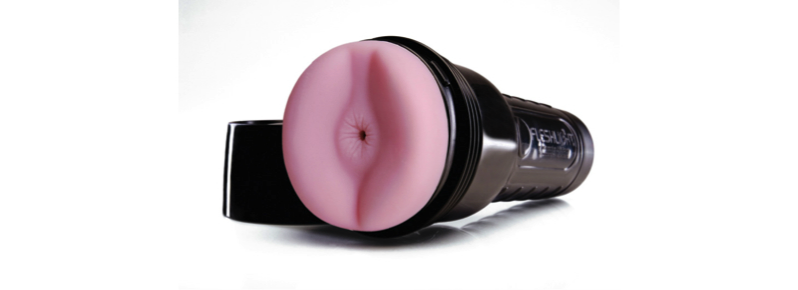
Anal Fleshlite used to demonstrate how to self-collect rectal swabs.

Interviewers use Fleshlite (Austin) models to demonstrate how to self-collect vaginal and rectal swabs (Figures 7 and 8) [36], while they use a mirror to locate the tonsils and demonstrate how to self-collect a pharyngeal swab.

Finally, we train interviewers on how to administer treatment for CT and NG infections. A physician prescribes the antibiotics, and interviewers are trained by the physician on how to properly deliver antibiotic therapy. Training includes information about antibiotic mechanisms, pharmacokinetics, potential adverse effects, partner therapy, retesting, and STI counseling. Interviewers practice providing treatment using sample scenarios to demonstrate competence.

### Testing Flow

While we perform HIV, CT, NG, and syphilis testing at every recruitment site, we only perform HCV testing at sites with populations at higher risk of HCV (history of incarceration or drug use). Every study participant receives every STI test unless they specifically choose to opt out. Opting out does not affect eligibility or reimbursement.

At baseline, the rapid HIV test is done as part of the eligibility screening to determine if the participant is HIV-infected or -uninfected. We determine eligibility based on a risk assessment, with a minimum risk score necessary for enrollment. Inclusion criteria and risk scoring are explained in detail in the ATN CARES protocol papers by Rotheram-Borus et al and Comulada et al [21,24]. If a participant is eligible for the study, they are enrolled and receive the additional STI testing. An interviewer with phlebotomy certification draws blood for use in the HIV RNA/DNA test, the syphilis test, and the HCV test at certain sites. The participant self-collects their urine sample or pharyngeal, rectal, and vaginal swabs. Clients are encouraged to stay until their test results are available.

Routine follow-up appointments occur at 4-month intervals for 2 years. However, if a patient reports potential STI exposure or STI symptoms to the interviewer, they will be invited for testing at any point during the study.

### Linkage to Care, Treatment, and Partner Management

Participants receive their test results on the same day as the testing. Whenever possible, they are told the results in person. If the participant needs to leave their appointment before results are available, the interviewer asks the participant how they would like to receive the results and then communicates the results with them through a phone call, text, or email. When a patient receives a positive test result, they are either referred to a partner medical clinic or to their primary care provider to receive treatment, or they are provided with treatment by the interviewing staff. All partner medical clinics agreed to and signed an STI treatment protocol that is in accordance with Centers for Disease Control and Prevention recommendations.

If the participant elects to seek treatment at a clinic, the interviewer works with the participant to find a clinic that is geographically convenient, and the study organizes free transport to the clinic via the Uber app. Interviewers counsel participants on the importance of partner treatment and safe sex practices. They also follow up with study participants to ensure they were able to receive treatment, and study staff obtains records of the treatment from the clinic.

For syphilis infections, we will always refer the participant to a clinical provider since follow-up blood work and a penicillin injection may be required. For CT and NG infections, it is up to the participant if they prefer to be referred to a clinic or receive same-day antibiotic treatment from the interviewing staff. The interviewers treat vaginal, urethral, and pharyngeal CT infections with 1 g oral azithromycin [1]. They treat rectal CT infections with 100 mg oral doxycycline twice daily for 7 days, as evidence shows doxycycline is more effective than azithromycin in treating rectal CT [37-40]. NG infection, while often clinically treated with 1 g oral azithromycin and an injection of 250 mg ceftriaxone, is instead treated with 1 g oral azithromycin and 400 mg oral cefixime [1]. The treatment of concurrent CT and NG infections are according to the type of CT infection. If there is a vaginal, urethral, or pharyngeal CT infection in addition to an NG infection, treatment is 1 g oral azithromycin and 400 mg oral cefixime. If there is a rectal CT infection in addition to an NG infection, treatment is 100 mg oral doxycycline twice daily for 7 days and 400 mg oral cefixime. All treatment regimens follow Centers for Disease Control and Prevention guidelines and are effective treatment methods.

We prepackage treatment packs that include antibiotic instructions, antibiotics, physician contact information, water, and a snack. Every recruitment site has these treatment packs available. We also offer participants with a positive CT or NG result up to 10 expedited partner therapy packets according to the number of partners reported in the past 90 days [41]. We provide the expedited partner therapy packs according to the type of infection participants test positive for as well as the type of sex they report having with their partner. For example, if a participant tests positive for urethral CT and reports insertive anal sex, we will provide doxycycline in the expedited partner therapy pack.

### Quality Control

The study team monitors STI prevalence to ensure that it falls within the expected range. A research assistant performs monthly quality control testing at every testing site to confirm that all tests are functioning properly. Monthly quality control testing involves running a positive and negative control sample for each rapid STI test at each site. This ensures that the tests correctly identify both positive and negative results. In addition to every month, we perform quality control testing whenever a new interviewer is conducting the tests, a new test lot number is received, or if the storage temperature falls outside the recommended range.

### Data Collection and Analysis

Interviewing staff record STI lab results on a paper lab form as well as through CommCare, a mobile data collection platform created by Dimagi (Cambridge). We then obtain the documentation of STI treatment from the medical clinics.

Using these data, we will evaluate STI prevalence, risk factors, and HIV seroconversion rates throughout the study period. We will also evaluate successful linkage to care and treatment of positive STI cases.

### Moving Forward

At the time of manuscript submission, we are in the process of making one change to our study protocol. Due to the high prevalence of a history of syphilis in our study population and the low specificity of the Syphilis Health Check, a participant with a positive Syphilis Health Check result requires additional laboratory testing. Therefore, we will obtain rapid plasma reagin titers and *Treponema pallidum* particle agglutination testing when a participant has a reactive Syphilis Health Check result. Quest Diagnostics will perform the rapid plasma reagin and *Treponema pallidum* particle agglutination tests. We anticipate that this change will significantly improve our ability to properly diagnose syphilis infections.

## Results

The project was funded in 2016, and enrollment will be completed in 2019. Preliminary data analysis is currently under way.

## Discussion

As STI prevalence in the United States continues to rise, it is critical to improve access to STI screening and treatment. This means improving the availability of acceptable and feasible screening methods, particularly for our country’s highest risk populations. In this study, we use point-of-care rapid diagnostic STI tests to screen adolescents for HIV, CT/NG, syphilis, and HCV. We are recruiting and enrolling participants at local community-based organizations in Los Angeles and New Orleans that cater to homeless youth and GBTY as well as youth with histories of drug use, mental health disorders, and incarceration. By targeting that traditionally tough-to-reach, high-risk group, we hope to determine the prevalence of STIs in the population and demonstrate the acceptability and feasibility of rapid STI testing and treatment programs in community-based settings.

A limitation of our study is that we are not evaluating cost-effectiveness. The GeneXpert machines used in our study were provided by the manufacturer as part of the Xpert CT/NG and Xpert HIV-1 Qual cartridge purchase agreement. While the machines themselves are expensive, they are cheaper than commercial laboratories. Moving forward, it would be advantageous to evaluate the cost-effectiveness of rapid STI testing in community-based settings.

## Abbreviations

ATN: Adolescent Medicine Trials Network
CARES: Comprehensive Adolescent Research and Engagement Studies
CLIA: Clinical Laboratory Improvement Amendments
CT: *Chlamydia trachomatis*
FDA: Food and Drug Administration
GBTY: gay, bisexual, and transgender youth
HCV: Hepatitis C virus
NG: *Neisseria gonorrhoeae*
STI: sexually transmitted infection
